# Whose pain is it anyway? Comparability of pain reports from children and their parents

**DOI:** 10.1186/s12998-016-0104-0

**Published:** 2016-08-01

**Authors:** Steven J. Kamper, Kristina Boe Dissing, Lise Hestbaek

**Affiliations:** 1 The George Institute for Global Health, University of Sydney, Sydney, Australia; 2 EMGO+ Institute, VU University Medical Centre, Amsterdam, The Netherlands; 3 Institute of Sports and Clinical Biomechanics, University of Southern Denmark, Campusvej 5, 5230 Odense M, Denmark; 4 Nordic Institute for Chiropractic and Clinical Biomechanics, Odense, Denmark

**Keywords:** Children, Adolescents, Parent, Pain report, Measurement, Musculoskeletal

## Abstract

**Background:**

There is a high demand for robust research into understanding the scope and consequences of musculoskeletal pain in children. An important part of this involves clarifying issues surrounding its measurement, not least differences in reporting from the children themselves and their parents. Therefore this study will assess the degree of agreement between parents’ report of their child’s pain and the child’s own assessment.

**Methods:**

Data were collected in 2013 and 2014 as part of a larger cohort study investigating the health of Danish school children. Two study samples included 354 and 334 child–parent pairs who were independently asked whether the child had experienced musculoskeletal pain in the previous week. Children were between the ages of 10 and 14 years old. Parents provided answers via text message and children were questioned in person or via questionnaire at their school.

**Results:**

Percentage agreement between parent and child assessment was around 50 % in Sample 1 and 68 % in Sample 2. The poor agreement was due to children reporting pain when their parent did not, the reverse very rarely occurred. Pain of greater intensity or longer duration resulted in better agreement between the child and parent. Child age and gender did not influence the likelihood of agreement.

**Conclusion:**

Children often experience pain that is not reported by their parents resulting in poor concordance between pain reports from the two sources. While it is not possible to say which is more valid we can conclude they are not interchangeable.

## Background

Published evidence points to the fact that musculoskeletal pain, and especially chronic pain in children and adolescents, is not only responsible for considerable personal suffering but also for a substantial economic burden [1]. In addition to its contemporaneous burden, pain in adolescence has also been shown to be an important predictor for pain in adulthood [2]. Musculoskeletal pain in children thereby represents a substantial societal problem, worthy of further investigation.

In comparison with the body of research pertaining to adults with musculoskeletal pain, there is a dearth of information relevant to children [3]. This is despite epidemiological evidence suggesting that prevalence rates for common conditions, such as back pain, in adolescents approach those in adults [4]. These factors point to the need for robust research into understanding the scope and consequences of pain in children. An important part of understanding more about pain in children and interpreting published research involves clarifying issues surrounding its measurement.

From a measurement perspective, the need to consider assessment of pain in children in a different manner to adults is well-accepted [5]. In recognition of this fact, the IMMPACT initiative considered their recommendations for the measurement of pain in children and adolescents separately from those for adults [6]. This is due to the different, and often rapidly evolving, physical, psychological and social factors that influence the report of pain.

Clinical assessment of children’s health conditions necessarily involves interaction with both children and their parents. The decision to seek care for a painful condition in pre-adolescent children typically rests with the parent(s) and further, once a decision to seek care has been made, parents are likely to be involved in the clinical encounter and have input into decisions about care. Thus while the child’s assessment holds a degree of inherent validity, the parents’ view is also relevant from a practical standpoint. In the context of epidemiological and clinical research into pediatric pain, a parent’s report is commonly used as a proxy for the child’s rating [7]. This is particularly relevant in longitudinal studies where children are too young to reliably self-report at the initial assessments although they might be able to do so at later follow-up points. It has been shown that the agreement between parent and child ratings is generally poor [8–10], although higher in the clinical samples than in the studies recruiting healthy children [11]. Nevertheless, reports of spinal pain in community or school based cohorts often fail to describe the degree of parents’ involvement in the data collection, lending uncertainty to the reported estimates [12]. Therefore, it is necessary to improve the understanding of the relationship between the two types of pain report, and draw attention to the importance of clear reporting.

### Aim

This study aims to explore the concordance between pain reports from parents and their children. It is hoped the findings of this study will aid interpretation of studies that have been conducted using parent report as a proxy for child’s pain and also inform design of future studies. The specific questions addressed in this study are as follows:

- How well do parents’ reports of the presence of musculoskeletal pain in their children match that of their children’s?
- Do gender, age, pain intensity and pain duration influence the likelihood that parental and child pain reports match?

## Methods

The analyses in the present study are based on two different samples, both nested within the CHAMPS study [7]. This study was designed to assess the effects of increased physical activity in school children in a municipality in Denmark. The CHAMPS study includes twice-yearly assessment of the children with numerous clinical measures and physical assessment.

The Regional Committees on Health Research Ethics for Southern Denmark approved the CHAMPS study (ProjectID: S-20110042).

### Parent’s pain report

Over the course of the CHAMPS study, parents were sent three SMS messages on a weekly basis asking simple questions about their child’s pain and sports participation over the past week. The average weekly response rate was 95 % over the two years prior to this study. Data from the following question was used in the present study:

A. “Has *“child’s name”* during the last week had any pain in:
  1. Neck, back or low back
  2. Shoulder, arm or hand
  3. Hip, leg or foot
  4. No my child has not had any pain.”

Parents replied to the text with the number in front of the correct answer.

### Children’s pain report

During the study period, children’s pain reports were collected in two different ways, one based on interviews and one based on questionnaires.

#### Sample 1

One of the assessment rounds of the main study was conducted during March and April 2013 and included approximately 1100 children. On the days where an extra test person from the research group was available, the children undergoing assessment were interviewed about their pain. Data from these interviews formed the child reports used in this study. Assessment included questions about the presence of spinal pain, upper limb pain and lower limb pain in the past week. If pain was present, it was located on a body chart divided into anatomical regions, pain intensity was assessed using an 11 point Numerical Rating Scale and duration was reported in response to the question: For how long have you had this pain? This was recorded in days. The children could report pain at more than one site, but the questions regarding intensity and duration related to the most intense pain.

#### Sample 2

As part of a validation process of the Young Spine Questionnaire, [13] 500 questionnaires were administered in the classroom in June 2014 to children who also partook in the CHAMPS study. The questionnaires were given to the teachers, who were asked to administer them at a convenient time. This questionnaire included the same questions for low back, mid back and neck pain separately. Using the neck questions as example, the first question was: “Have you ever had pain in your neck?” (response options: “often”/“sometimes”/“once or twice”/“never”). If pain was reported, the next questions were: “Have you had neck pain in the last week?” (“yes”/”no”) and “Do you have neck pain today?” (“yes”/”no”). Next, they noted the worst pain ever in the neck using the revised version of the Faces Pain Scale (FPS-R) [14]. This scale is based on six faces with expressions illustrating progressively worse pain. The questions were repeated for the mid back and low back. A diagram with the three spinal areas clearly shaded and labelled was shown alongside the questions. For this study, the weekly prevalence and pain intensity were used. All three regions were collapsed into the variable ‘spinal pain’, and if pain was present at more than one site, the highest intensity was used. Furthermore, as part of the YSQ, they were asked about consequences of their spinal pain: “Have you stayed home from school because of neck or back pain?”, “Has neck or back pain sometimes stopped you from doing sports?” and “Have you been to a doctor, chiropractor or physiotherapist because of neck or back pain?”

### Analysis

Concordance of information from the assessment of the child and SMS responses of the parents was calculated. Parents’ SMS reports of the presence of pain were gathered from responses to two consecutive SMS messages that fully encompassed the week prior to the date of the child’s pain report. This was necessary because physical assessments/questionnaire administration and SMS messages were not on the same day and it ensured that the time period reported by the parent (the two weeks up to the second SMS) completely overlapped that of the child (the week up to the day of the interview). Due to differences in the data collection methods between the two samples data were analyzed separately.

For Sample 1, cross-tabulations were presented to assess the concordance of a report of any pain and of pain specific to one of the defined regions; spine, upper limb, or lower limb, for Sample 2 only for spinal pain. Concordance between parent and child report was expressed as percentage agreement and kappa values, including prevalence and bias adjusted Kappa values. All child–parent dyads were coded as concordant or discordant for the analyses investigating the influence of the various factors on concordance.

The influence of gender was presented by constructing a cross-tabulation and performing a chi-squared test for the difference in proportions. Mean pain intensity (both samples) and pain duration (Sample 1) for the concordant and discordant cases was calculated and compared via independent samples t-tests. Since questions regarding pain intensity and duration were not relevant for children without pain, these cases were removed for those comparisons. Investigations of the influence of age (grade year) were conducted as per that for pain intensity, except that the whole sample was used i.e. including those concordant for ‘no pain’. All analyses were performed in SPSS 20.

## Results

### Study sample

Sample 1 included 354 children in grades 4 (approximate age 10 yr), 5, 6, 7 and 8 (approximate age 14 yr), the balance of boys and girls was close to equal (52.7 % female), and there were proportionally more students in the younger classes than in the older. Sample 2 included 334 children in grades 5 to 8 with 54.8 % females, they were slightly older on average than the children in Sample 1 (Table 1). Some teachers did not find classroom time to administer the questionnaires and therefore, only 67 % were distributed. All questionnaires actually administered to the children were returned.

**Table 1:** Characteristics of sample

	Sample 1		Sample 2	
Gender; number (%)	Male	Female	Male	Female
	166 (47.3)	185 (52.7)	151 (45.2)	183 (54.8)
Age; number (%)				
Grade 4 (10 yr)	83 (23.4)		-	
Grade 5 (11 yr)	80 (22.6)		84 (25.1)	
Grade 6 (12 yr)	77 (21.8)		125 (37.4)	
Grade 7 (13 yr)	54 (15.3)		47 (14.1)	
Grade 8 (14 yr)	60 (16.9)		78 (23.4)	
Pain Report; number (%)	Child	Parent	Child	Parent
Spine	132 (37.0)	26 (7.8)	126 (37.7)	21 (6.6)
Upper limb	37 (10.4)	11 (3.3)	-	-
Lower limb	126 (35.3)	45 (13.6)	-	-
Any pain^a^	237 (66.4)	74 (22.3)	-	-
Pain Intensity; mean (SD)	5.47 (2.17)^b^		2.87 (1.19)^c^	
Pain duration in days; mean (SD)	77.77 (246.4)			
Impact of pain				
Missed school			20.8 %	
Missed sport			54.8 %	
Consult healthcare prof.			56.8 %	

^a^Any pain: spine pain or upper limb pain or lower limb pain

^b^0–10 Numerical rating scale

^c^0–5 Faces pain scale

### How well do parents’ reports of their children’s pain match those of their children’s?

Reports of pain from parents and their children generally showed quite poor concordance. With respect to the presence or absence of pain, regardless of region; percentage agreement was 52.4 % and kappa 0.20 (95 % CI, 0.14 to 0.26) in Sample 1, and 68.4 %, and 0.19 (0.12 to 0.27), respectively, in Sample 2. Prevalence-adjusted and bias-adjusted kappa statistics were also calculated and were 0.05 for Sample 1 and 0.37 for Sample 2. Notably, parents very rarely reported pain when the child did not; the low agreement is almost entirely due to children reporting pain and the parent not (Table 2).

**Table 2:** Concordance of report of pain

		Sample 1 (any MSK pain)			Sample 2 (spinal pain)		
		Parent			Parent		
		Pain	No pain		Pain	No pain	
Child	Pain	70	154	224	20	100	120
	No pain	4	104	108	1	199	200
		74	258	332	21	299	320

In Sample 1, where questions were asked about MSK pain in the whole body, agreement was inevitably poorer when concordance of pain report specific to a body region was assessed; agreement was 48.8 % although the kappa value was slightly higher, 0.26 (95 % CI, 0.19 to 0.32). These data indicate that while parents often report that their child has no pain when the child reports that he/she does, there is less commonly discordance regarding the site of pain (Table 3). When parents did report pain, the agreement regarding the site of pain was 78 %.

**Table 3:** Concordance specific to region – Sample 1

		Parent				
		Spinal pain	UL pain	LL pain	No pain	
Child	Spinal pain	21	1	6	87	115
	UL pain	0	6	3	17	26
	LL pain	1	1	31	50	83
	No pain	2	0	2	104	108
		24	8	42	258	332

*UL* upper limb, *LL* lower limb

In some cases the parent and/or the child reported pain at more than one site, when this occurred, the cases were considered concordant if one of the sites was reported by both parent and child. For the purpose of the results presented in Table 3, the case was coded for this (matching) site only.

### Do gender, age, pain intensity and pain duration influence the likelihood that parental and child pain reports match?

The proportion of girls that reported pain (Sample 1 42.7 %, Sample 2 38.8 %) was slightly higher than the proportion of boys (31.3 %, 36.4 %). There was no statistically significant effect of gender on the likelihood of concordance between parent and child pain report (Table 4). There was no overall difference in mean age of the child between the concordant parent–child pairs and the discordant pairs (Table 5).

**Table 4:** Concordance according to gender

	Sample 1		Sample 2	
	Boys	Girls	Boys	Girls
Concordant	67/150 (44.7 %)	93/180 (51.7 %)	99/141 (70.2 %)	120/179 (67.0 %)
Discordant	83/150 (55.3 %)	87/180 (48.3 %)	42/141 (29.8 %)	59/179 (33.0 %)
	Pearson *χ*2 1.61, *p* = 0.21		Pearson *χ*2 0.37, *p* = 0.55

**Table 5:** Concordance according to age, pain intensity and pain duration

	Concordant	Discordant	Mean Difference	*p*-value
Age (grade)				
Sample 1	5.88 (1.4) *n* = 162	5.73 (1.4) *n* = 170	0.15	0.32
Sample 2	6.26 (1.1) *n* = 219	6.55 (1.1) *n* = 101	0.29	0.03
Intensity				
Sample 1: MSK^a^	6.40 (1.7) *n* = 58	5.16 (2.2) *n* = 170	1.24	<0.01
Sample 2: Spinal^b^	3.15 (1.15) *n* = 20	2.81 (1.15) *n* = 100	0.34	0.23
Duration (days)				
Sample 1	129 (366) *n* = 52	62 (193) *n* = 164	67	0.09

^a^0–10 Numerical rating scale

^b^0–5 Faces pain scale

Analyses investigating whether there was a difference in pain intensity and pain duration between concordant and discordant pairs were conducted after excluding the cases that were concordant for no pain. In the data from Sample 1 there was a statistically significant difference in mean pain scores in concordant versus discordant pairs; this difference indicates that parents are more likely to report pain that is of greater intensity. There was no significant difference in the mean pain scores on the faces pain scale in the concordant versus discordant groups in Sample 2. While there was quite a large difference in mean pain duration (67 days), the very large standard deviations ensured it was not statistically significant (only measured in Sample 1).

## Discussion

The concordance between parents’ and their child’s report of pain was generally quite poor. Children very often reported pain that was not reported by their parents, whereas the reverse was very rarely the case. When the parents did report pain, the agreement with the child regarding the site of pain was reasonably good, at close to 80 %. While it may be that concordance is better for some pain sites than others, the sample size was not large enough to investigate these differences.

The relationship between pain intensity and concordance, which reached statistical significance in the interviewed sample (Sample 1), reflects the unsurprising finding that parents were more likely to report their child’s pain when it was of greater severity. There were also differences in terms of pain duration between the concordant parent–child pairs and the discordant pairs in Sample 1. While this difference did not reach statistical significance, it was quite large (67 days) and in the expected direction i.e. better concordance for longer term pain. There was no influence of child gender or age.

Relevant research exploring the relationship between children’s and parents’ pain report comes from population based studies and from studies conducted on clinical populations. The latter generally include children experiencing acute surgical or procedural pain, or receiving medical care for a chronic condition. Population based studies are more directly comparable with the results of our study while the clinical studies often offers more in-depth insight into the issue in specific populations. In general, agreement between parent and child ratings is higher in the clinical samples than in the studies recruiting healthy children [11].

Chiwaridzo and colleagues [15] recently conducted a study addressing a similar question to ours in adolescent school-children and their parents in Zimbabwe. The level of agreement they report (kappa; 0.2) was very similar to that observed in our study, they also noted that the discordance was due to parents reporting no pain when their child reported pain. These similar findings provide support for the generalizability of these results, across countries with different socio-economic profiles. Kroner-Herwig et al. [9], Haraldstad et al. [8], Sundblad et al. [10] and Waters et al. [16] all report on community-based surveys of children and investigated the concordance between parents’ and children’s pain report. While the children filling in the questionnaires in these studies report a range of different types of pain e.g. musculoskeletal, headache, stomach-ache etc., overall the results from these studies are broadly similar to those of ours. Three of the four studies [8–10] reported generally poor agreement between parent and child ratings, while the last reported somewhat higher concordance [16]. Three studies [9, 10, 16] also found that children reported pain more often (and of greater intensity) than their parents, in the fourth study this relationship held for girls aged 12 and over but not for boys or younger girls. The influence of age and gender on concordance of the ratings was variable across the studies, but the finding that agreement between the ratings was better where the condition was more severe was reported in all studies, as in the present study.

Zhou and colleagues [17] performed a systematic review and meta-analysis of nine studies relating to the relationship between parents’ and children’s pain ratings and calculated a pooled correlation of 0.64. Studies included in the review recruited children experiencing pain due to surgery or medical procedures. Vetter [18], studying patients attending an outpatient chronic pain clinic and Cohen [11] on patients from rheumatology or pain treatment programs found moderate to good concordance between ratings of Health-related Quality of Life and physical functioning respectively. Counter to the findings of the population-based studies, in both cases parents tended to over-report severity of the impacts on their children. It is possible that the better concordance and switching from under- to over-reporting of pain reflects the greater sensitivity and concern paid by parents of sick children. Alternatively, the nature of concordance between measures of quality of life and physical function may be fundamentally different to that of pain.

In recent decades the ideal of ‘patient-centered practice’ has achieved increasing prominence. Central to this is the primacy of the patient’s own experience of their condition, treatment and outcome [19]. If we are to apply this principle to the results of our study, we assume that the child’s viewpoint is inherently valid, and may conclude that the parent’s rating is an unreliable substitute. However, the nature of the relationship, in particular the fact that parents almost never report pain when the child does not, invites further speculation. It is possible that parents are unaware of the minor aches and pains experienced by their children, or that they may apply a kind of ‘filter’, whereby they attend only to pain that they consider significant or meaningful. This conclusion is at least partly supported by the finding that higher pain intensity was associated with better concordance. If this is the case it would be of interest to know whether parent-reported, or child-reported pain served as a more useful indicator of more serious, ongoing, or future health conditions. Exploration thus of the predictive or prognostic validity of the two types of pain report could be a worthwhile target for further research.

From a clinical point of view, it can be said that parents’ report of their child’s pain is not likely to provide a reliable assessment of the child’s experience. Care should be taken though when generalizing this finding beyond the healthy population, since concordance may be better in groups of children receiving medical care for a painful condition.

Similarly the distinction between the findings from population based and clinical studies should be kept in mind when comparing the results from different studies. We can conclude based on findings from this, and previous studies, that in healthy populations parents’ report of their children’s pain is likely to underestimate the pain experience of the children. Obviously, the ‘best’ measure depends on the particular research question, but it is clear that the two measures should not be considered interchangeable. As regards to future research, exploration of the validity (e.g. concurrent, predictive) of the two types of report would inform decisions regarding whether one or the other is best suited to a particular research question.

This study has several strengths; it involves data collected from a representative sample of children attending schools in a western European country (Denmark). The data completeness is acceptable (<7 % missing data) and was collected within a large observational study, data were collected in two different ways (interview and questionnaire) and inclusion was based on availability of interviewers for Sample 1 and classroom time for Sample 2, and thus not subject to selection bias.

There are however some limitations, chief among which is the fact that mode of pain report was different for the parents and the children. Parents reported pain via SMS and children by answering questions in a face-to-face interview with a researcher or by answering a written questionnaire. The impact of this limitation is that part of the discordance observed in the study could be due to different data collection methods which would mean the real level of agreement between children and their parents is somewhat better than our findings indicate. The fact that this limitation is responsible for a systematic, rather than random bias means we can factor it in to our interpretation, that is to say that the concordance we observe in this study represents a minimum estimate of agreement between children and their parents regarding the existence of pain. The study also lacked power to explore detailed questions regarding reporting of pain at different sites and between age groups. Finally, since parents and children were not asked on the same day, there might be complaints reported by the parents which occurred after the interviews with the children. However, even if this occurred it would not alter the main conclusions of the study.

## Conclusion

A parent’s report of the presence of pain in their child does not correspond well with the child’s own report in healthy children from 10 to 14 years of age. Children commonly report pain that is not reported by their parent, whereas the reverse is rarely the case. Not unexpectedly, parents and children are more likely to agree on the presence of pain if the pain is more intense and possibly if the pain is of a longer duration. Agreement is not influenced by age or gender of the child. Whether the one measure is more suitable than the other will depend on the particular research question, but it is clear that the two measures should not be considered interchangeable.
